# CATALYZING DEMENTIA CARE THROUGH THE LEARNING HEALTH SYSTEM AND CONSUMER HEALTH INFORMATION TECHNOLOGY

**DOI:** 10.1093/geroni/igad104.0573

**Published:** 2023-12-21

**Authors:** Jennifer Wolff, Danielle Powell

## Abstract

To advance care for persons with Alzheimer’s disease and related dementias (ADRD), real-world health system effectiveness research must actively engage those affected to understand what works, for whom, in what setting, and for how long – an agenda central to Learning Health System principles. The spread of consumer health information technology affords new opportunities to support ADRD caregivers within health care delivery. Patient portals allow patients to view test results and sections of their electronic medical record, perform health management tasks (e.g., scheduling visits, billing, filling prescriptions), access educational materials, and communicate with clinicians using secure messaging. Many care delivery organizations allow patients to share access to their portal account by registering a “care partner” to have their own identity credentials (login/password). Through shared access, patients may select whether and who electronically interacts with clinicians on their behalf, thus, respecting patient preferences while supporting care partners with timely information about patient health and treatments, and a mechanism to electronically navigate health system demands. Little is now known about electronic information sharing preferences and practices of persons with ADRD and their caregivers, the feasibility of deploying novel interventions, or existing initiatives through consumer health information technologies to better support ADRD care and management. This symposium presents early results from projects seeking to increase evidence regarding the role and use of consumer health information technology in ADRD care and management by examining the existing landscape of patient portal use in the context of dementia care from a large integrated academic health system.

